# The complete chloroplast genome of *Lantana camara* L. (Verbenaceae)

**DOI:** 10.1080/23802359.2020.1719920

**Published:** 2020-01-27

**Authors:** Samaila S. Yaradua, Muzammil Shah

**Affiliations:** a Centre for Biodiversity and Conservation, Department of Biology, Umaru Musa Yaradua University, Katsina, Nigeria;; b Department of Biological Sciences, King Abdulaziz University, Jeddah, Saudi Arabia

**Keywords:** Chloroplast genome, Illumina sequencing, *Lantana camara*, phylogenetic analysis

## Abstract

In this study, we sequenced, assembled and reported the complete chloroplast genome of *Lantana camara* an important medicinal plant for the first time. The genome is circular and quadripartite in structure; it has a length of 154,388 bp, 39.2% GC content and harbored 137 genes including 90 protein-coding genes, 39 tRNAs, and 8 rRNAs. The genome contained a large single-copy of 85,198 bp and a small single-copy of 17,249 bp separated by a pair of inverted repeat regions. The phylogenetic relationship showed a close relationship between *L. camara* and *Lippia origanoides.* The plastome sequence reported in this study will help for future research on the species and evolutionary studies of Verbenaceae.

*Lantana camara* L. is a member of the Verbenaceae family; the species is native of Neotropics and is cultivated widely for its ornamental and medicinal values (Srivastava et al. 2005). The plant is an evergreen shrub and grows up to 4 m tall with dense thickets (Day et al. 2003). *Lantana camara* is widely used in traditional medicine, especially in Saudi Arabia (Khan et al. 2016), and is reported to contain the following compounds: flavonoids, triterpenoids, steroids, oligosaccharides, phenylpropanoid glycosides, and naphthoquinones (Begum et al. 2014). Additionally, *L. camara* is used in the treatment of asthma, measles, tetanus, fever, eczema, and tumors (Sathish et al. 2011). Several studies have been conducted on phytochemistry and the medicinal properties of the plant. Until this study, no research was conducted on the genome of the plant despite its medicinal potentials. A complete chloroplast (cp) genome is a tool that provides needful information for taxonomic studies, tracing source population and tracing of phylogenetic relationships to resolve taxonomic problems (Parks et al. 2009). This is as a result of the conservative nature of the cp genome, low substitution rate, and maternal inheritance. Despite the importance of the plastome sequence in taxonomic and evolutionary studies, the cp genome of *L. camara*, a medicinally important plant, is not reported. In this study, we reported the complete cp genome of *L. camara*, the third to be sequenced in the Verbenaceae family.

The plant was grown in the garden of the department of biological sciences, King Abdulaziz University, Jeddah (39°15′0.60′′E, 21°29′22.79′′N). Fresh leaves of *L. camara* were collected for DNA extraction and sequencing. A voucher specimen was prepared and deposited in the herbarium of King Abdulaziz University, Jeddah with the accession number KAU27361. Total genomic DNA was extracted using a Qiagen DNA extraction kit (Qiagen, Hilden, Germany) based on the manufacturer’s protocols. The genomic DNA was sequenced using Illumina Hiseq 2500 platform (Novogene Technology, Inc., Beijing, China). Raw data were filtered using PRINSEQ lite Ver0.20.4 to obtain clear reads (5 GB). Clean reads sequenced were assembled with NOVOPlasty2.7.2 (Dierckxsens et al. 2017). The assembled sequenced were annotated using DOGMA (Wyman et al. 2004) followed by manual adjustment using BLAST (https://blast.ncbi.nlm.nih.gov/Blast.cgi), trNAscan-SE2.0 (Lowe and Chan 2016) was used to identify tRNA genes. Finally, the complete cp genome sequence of *L. camara* was submitted to the Genbank with accession number MK416153.

The complete plastome sequence of *L. camara* is circular and quadripartite in structure and has a length of 154,388 bp, the cp genome has 39.2% GC content and consist of 137 genes including 90 protein-coding genes, 39 tRNA, and 8 rRNA genes, a large single-copy (LSC) region of 198 bp, a small single-copy (SSC) region of, 249 bp and a pair of inverted repeat regions (IRa and IRb) of 25,971 bp each.

To confirm the phylogenetic positions of *L. camara* in the Verbenaceae, the complete cp genome of species in the Verbenaceae available in Genbank, namely *Lippia origanoides* (MK248831.1) and *Aloysia citrodora* (KY085903.1), were downloaded. *Premna microphylla* (NC_026291.1) and *Scutellaria baicalensis* (MF521633.1) of the Lamiaceae family were also downloaded from the Genbank to be used as outgroup. The cp genome sequence of *L. camara* and that of the downloaded sequences were aligned with MAFFT and analyzed using Bayesian analysis. The result of the phylogenetic analysis showed that Verbenaceae is monophyletic and *L. camara* is a sister taxon to *L. origanoides* (Figure 1).

**Figure 1.**
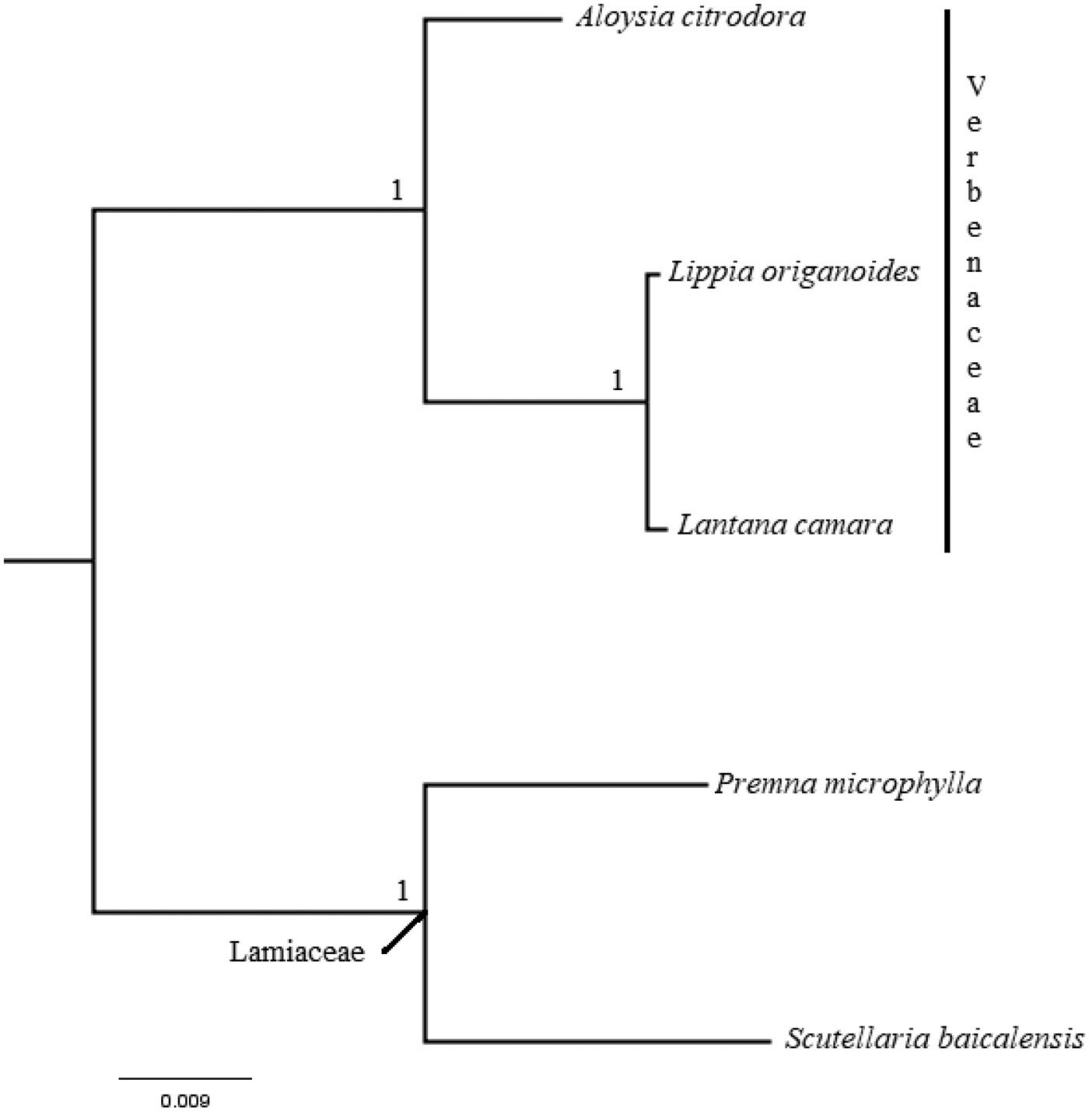
Bayesian Inference phylogenetic tree of *Lantana camara* with other Verbenaceae species based on complete chloroplast genome sequence. Numbers in the nodes represent posterior probability (PP) values.

## References

[CIT0001] Begum S, Ayub A, Qamar Zehra S, ShaheenSiddiqui B, IqbalChoudhary M. 2014. Leishmanicidaltriterpenes from *Lantana camara*. Chem Biodivers. 11:709–718. 24827681 10.1002/cbdv.201300151

[CIT0002] Day MD, Wiley CJ, Playford J, Zalucki MP. 2003. Lantana: current management status and future prospects. Canberra (Australia): Australian Centre for International Agricultural Research.

[CIT0003] Dierckxsens N, Mardulyn P, Smits G. 2017. Novoplasty: de novo assembly of organelle genomes from whole genome data. Nucleic Acids Res. 45(4):e18. 28204566 10.1093/nar/gkw955 PMC5389512

[CIT0004] Khan M, Mahmood A, Alkhathlan HZ. 2016. Characterization of leaves and flowers volatile constituents of *Lantana camara* growing in central region of Saudi Arabia. Arab J Chem. 9(6):764–774.

[CIT0005] Lowe T, Chan P. 2016. tRNAscan-SE on-line: integrating search and context for analysis of transfer RNA genes. Nucleic Acids Res. 44(W1):W54–W57. 27174935 10.1093/nar/gkw413 PMC4987944

[CIT0006] Parks M, Cronn R, Liston A. 2009. Increasing phylogenetic resolution at low taxonomic levels using massively parallel sequencing of chloroplast genomes. BMC Biol. 7(1):84. 19954512 10.1186/1741-7007-7-84 PMC2793254

[CIT0007] Sathish R, Vyawahare B, Natarajan K. 2011. Antiulcerogenic activity of *Lantana camara* leaves on gastric and duodenal ulcers in experimental rats. J Ethnopharmacol. 134(1):195–197. 21129476 10.1016/j.jep.2010.11.049

[CIT0008] Srivastava SK, Khan M, Khanuja SPS. 2005. Process for isolation of hepatoprotective agent “oleanolic acid” from *Lantana camara.* U.S. Patent 6,884,908.

[CIT0009] Wyman S, Jansen R, Boore J. 2004. Automatic annotation of organellar genomes with DOGMA. Bioinformatics. 20(17):3252–3255. 15180927 10.1093/bioinformatics/bth352

